# Communication is key: Innate immune cells regulate host protection to helminths

**DOI:** 10.3389/fimmu.2022.995432

**Published:** 2022-09-26

**Authors:** Jianya Peng, Hannah G. Federman, Christina M. Hernandez, Mark C. Siracusa

**Affiliations:** ^1^ Center for Immunity and Inflammation, New Jersey Medical School, Rutgers-The State University of New Jersey, Newark, NJ, United States; ^2^ Department of Medicine, New Jersey Medical School, Rutgers-The State University of New Jersey, Newark, NJ, United States

**Keywords:** innate immune cells, neuro-immune crosstalk, innate immunity, antihelminth immunity, host protection

## Abstract

Parasitic helminth infections remain a significant global health issue and are responsible for devastating morbidity and economic hardships. During infection, helminths migrate through different host organs, which results in substantial tissue damage and the release of diverse effector molecules by both hematopoietic and non-hematopoietic cells. Thus, host protective responses to helminths must initiate mechanisms that help to promote worm clearance while simultaneously mitigating tissue injury. The specialized immunity that promotes these responses is termed type 2 inflammation and is initiated by the recruitment and activation of hematopoietic stem/progenitor cells, mast cells, basophils, eosinophils, dendritic cells, neutrophils, macrophages, myeloid-derived suppressor cells, and group 2 innate lymphoid cells. Recent work has also revealed the importance of neuron-derived signals in regulating type 2 inflammation and antihelminth immunity. These studies suggest that multiple body systems coordinate to promote optimal outcomes post-infection. In this review, we will describe the innate immune events that direct the scope and intensity of antihelminth immunity. Further, we will highlight the recent progress made in our understanding of the neuro-immune interactions that regulate these pathways and discuss the conceptual advances they promote.

## Introduction

**Figure 1:**
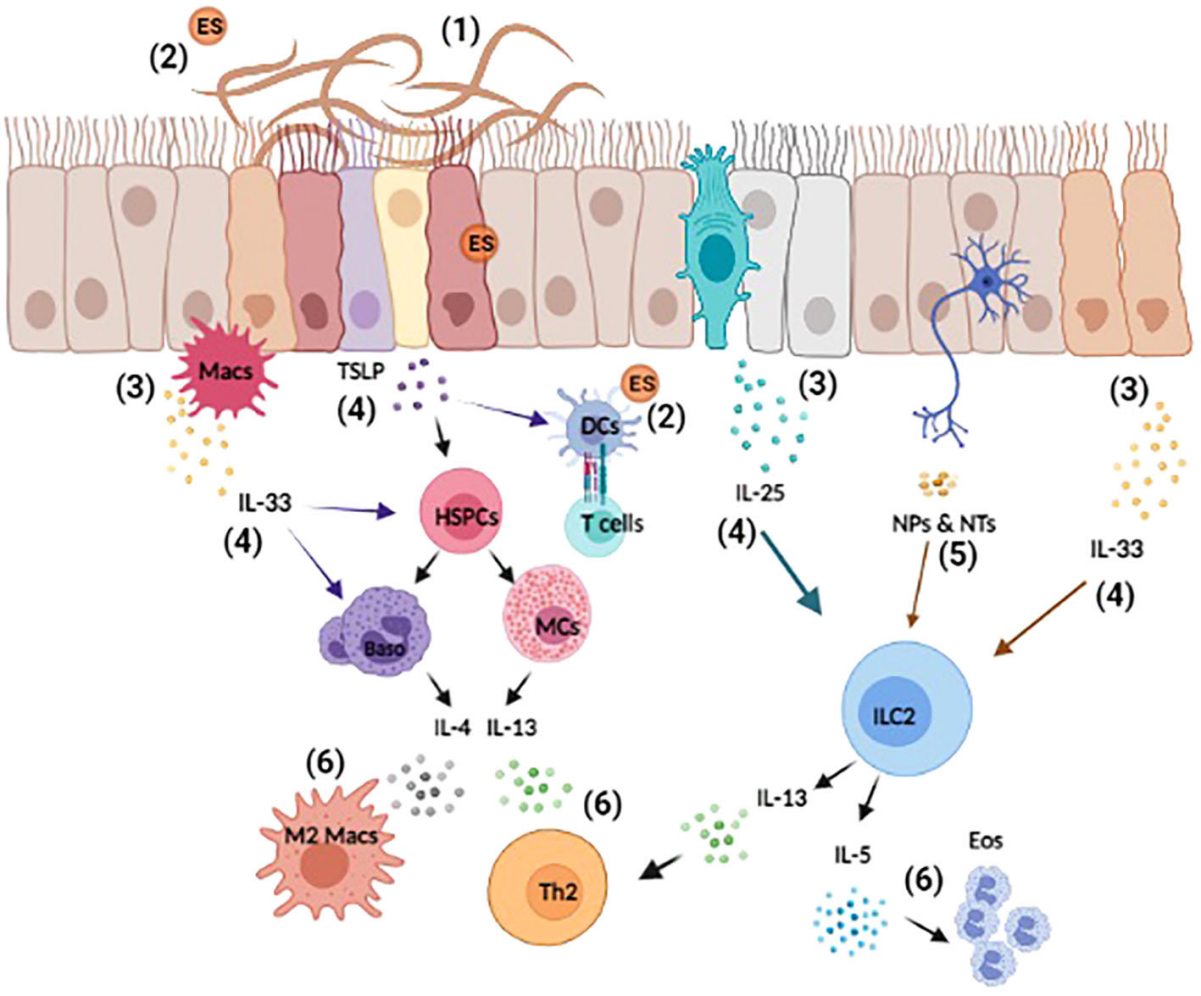
Overview of helminth-induced innate immune responses. Upon invasion, helminths cause substantial tissue damage as they burrow through various barriers and organs, such as the lungs, gut, and skin (1). Helminth also release excretory-secretory (ES) products that can act both locally and systemically (2). The damage-associated and helminth-derived signals promote the production of cytokine alarmins (IL-25, IL-33, and TSLP) from both hematopoietic and non-hematopoietic cells, such as macrophages (Macs) and epithelial cells (3). These early events drive the activation and expansion of innate immune cells, hematopoietic stem/progenitor (HSPCs), mast cells (MCs), basophils (Baso), dendritic cells (DCs), and ILC2s etc. (4). Moreover, innervating neurons can respond to helminth-derived signals by producing neuropeptides (NPs) and neurotransmitters (NTs) that directly influence immune cell activation and regulate inflammation (5). Collectively, these events induce the production of the type 2 cytokines IL-4, IL-5, and IL-13 that promote the polarization of type 2 T helper (T_H_2), the induction of M2 macrophages and eosinophilia (6). Reviewed in (9, 23, 41–43).

Since Norman Stoll’s hallmark paper 75 years ago revealing the global burden of helminthiasis, various systemic- and meta-analyses have demonstrated that more than a quarter of the global population is infected with helminth parasites (1–5). Included among these infections are nematodes that can be categorized as roundworms (*Ascaris lumbricoides*, *Trichinella spiralis*, and *Strongyloides stercoralis*), whipworms (*Trichuris trichiura*) and hookworms (*Ancylostoma duodenale* and *Necator americanus*). Additionally, helminths are also comprised of platyhelminths, or flatworms, that include free-living turbellarian flatworms, land planarians, and the disease-related Neodermata, consisting of both flukes (schistosomes) and tapeworms (6). These diverse parasites can be transmitted by the consumption of food or water that is contaminated with eggs, via insect bite, or by the parasites directly penetrating the skin (4, 5). Despite their prevalence, helminth infections have long been considered as neglected tropical diseases (NTD) that result in malnutrition, significant morbidity, growth retardation, cognitive deficiencies, and immunopathology (3, 7–9). Control of these NTDs mainly relies on mass antihelmintic drug administrations (MDA) with compounds such as albendazole or mebendazole to reduce worm burdens. These treatments are often combined with improved sanitation measures to prevent future infections (10). Despite these efforts, reinfection rates remain extremely high, with studies showing that up to 60% of individuals can be reinfected within 6-12 months of receiving treatment (3, 10). The frequent use of MDA has also resulted in an increased risk of drug-resistant helminths, a trend that is already seen in livestock populations (11). These limitations highlight the significant need for the development of more dependable and enduring treatment strategies, such as effective immunotherapies. Unfortunately, the development of immune-based therapies has been fraught with difficulty due to the complexity of helminth life cycles and their stage-dependent antigenic variation (10, 12). Further, our incomplete understanding of how antihelminth immunity is initiated and regulated has proven to be another substantial hurdle. To address this, many groups have sought to better understand the innate immune events that promote host protective responses to helminths. Given that investigating helminth infections in patient populations is extremely challenging, many studies have employed animal models infected with *Trichuris muris*, *Trichinella spiralis*, *Nippostrongylus brasiliensis*, *Heligmosomoides polygyrus*, *Strongyloides ratti*, *Strongyloides venezuelensis*, *Brugia malayi*, and *Schistosoma mansoni* to study the mammalian immune response to these parasites [summarized in **Table 1** (13–18), also reviewed by (20, 21)]. Collectively, these animal models have tremendously informed our understanding of the innate immune responses activated upon the initial exposure to these parasites (22, 23). As mentioned above, antihelminth immunity is primarily mediated by type 2 cytokine responses that are characterized by the development of type 2 helper T (T_H_2) cells. During a helminth infection, it is well appreciated that inflammation is initiated by the release of specific cytokines from immune cells and epithelial cells, such as, Tuft cells at barrier surfaces (24–29). Included among these rapidly released molecules are interleukin (IL)-25, IL-33, and thymic stromal lymphopoietin (TSLP) (30–32) that are produced in response to both the physical damage caused by the worms and also their release of excretory-secretory (ES) products (24, 26, 27, 33, 34). The production of these effector molecules mobilizes and activates diverse populations of innate immune cells that help to promote the development of T_H_2 cells (30, 35). Once activated, T_H_2 cells produce IL-13, influencing goblet cells within infected epithelial barriers to increase mucus production and facilitate worm expulsion (36, 37). Moreover, IL-13 from IL-25 activated ILC2 can regulate epithelial cell differentiation and drive a more secretory epithelial phenotype to facilitate intestinal remodeling and worm expulsion (33, 38). At the same time, T_H_2 cells produce IL-4 and IL-5 to promote the population expansion of alternatively activated (M2) macrophages and the migration of eosinophils to the affected tissues (39). Collectively, this cascade of events serves to clear worms, while also promoting wound healing once the worms are killed or expelled (40) (Summarized in **Figure 1**).

**Table 1:** Experimental animal models of helminth infections.

Animal model	Experimental route of inoculation	Infection stage	Natural route of infection	Affected compartment^1^	Human pathogen equivalent	Population affected annually worldwide estimated
***Trichuris muris***	p.o.	Eggs	Oral ingestion	Intestinal tract	*Trichur* ***i*** *s trichiura*	~ 800 million (13)
***Trichinella spiralis***	p.o.	L1 larvae	Oral ingestion	Intestinal tract, skeletal muscle	*Trichinella spiralis*	~ 10000 cases (14)
***Heligmosomoides polygyrus***	p.o	L3 larvae	Oral ingestion	Intestinal tract	*Ascaris lumbricoides, Ancylostoma duodenale, Necator americanus*	*A. Lumbricoides*: 807 million–1.2 billion (15); *A. duodenale* & *N. americanus*: ~ 740 million (16);
***Nippostrongylus brasiliensis***	i.d., s.c.	L3 larvae	Skin penetration	Skin, lungs, intestinal tract		
***Strongyloides ratti***	s.c.	L3 larvae	Skin penetration	Skin, intestinal tract	*Strongyloides stercolaris*	30~ 100 million (17)
***Strongyloides venezuelensis***						
***Litomosoides sigmodontis***	s.c., mite	L3 larvae	Mosquito Blackflies	Blood, pleural cavity	*Brugia malayi, Wuchereria bancrofti, Onchocerca volvulus*	~ 90.2 million (18)
***Schistosoma mansoni***	Percutaneous exposure s.c.	Cercariae	Skin penetration	Skin, liver, lungs	*Schistosoma* spp.	~ 200 million (19)

^1^Affected compartment: refers to the experimental models of infection. Intestinal tract changes generally include both host and commensal alterations that occur across both the small and large intestines.

While many non-hematopoietic and adaptive immune cells play critical roles in the promoting host protection to helminth, this article will highlight important studies that have substantially increased our understanding of the various innate immune cells that initiate and regulate helminth-induced inflammation. We will first review recent reports demonstrating the crucial roles various myeloid cells play in promoting host protection to helminths. We will then emphasize the known contributions of innate lymphoid cells to antihelminth immunity. Finally, we will discuss emerging studies defining how these pathways can be regulated by neuro-immune communication occurring within the affected tissue and highlight how neuro-immune crosstalk appears to fine-tune antihelminth immunity to promote optimal outcomes (clearing the worms and restoring tissue homeostasis).

## The contributions of mast cells to antihelminth immunity

Mast cells (MCs), having matured from progenitors in peripheral tissues, seed barrier surfaces and are ideally suited to respond to invading helminths. Once activated, MCs are well described for their ability to influence the development and persistence of T_H_2 cell-associated responses via their release of effector molecules including histamines, leukotrienes, prostaglandins, cytokines, and proteases (44, 45). Specifically, MCs can assist with the polarization of T_H_2 cells, in part, through the secretion of IL-4 and IL-13 (45). MCs can also influence the activation of antigen presenting cells (APCs), and some studies suggest that mast cells can act as APCs and thereby directly influence T-cell responses. However, the APC functions of MCs are debated and have been challenged by other reports (46–48). The below sections will summarize the known contributions of MCs to type 2 cytokine-mediated inflammation and antihelminth immunity.

Mastocytosis is an established feature of helminth-induced inflammation (49–53) but the roles MCs play in promoting antihelminth immunity are still being elucidated (54–57). While the precise mechanisms that govern the expansion of MC populations remain to be fully defined, studies have reported that mast cell precursors respond to helminth-induced alarmins such as IL-25, IL-33, and TSLP (34). Specifically, IL-25 has been shown to induce a population of c-Kit-expressing progenitor cells that possess MC potential and support immunity to *Trichuris muris* (58–60). MC populations also expand and activate in response to cytokine alarmins following infection with *Heligmosomoides polygyrus bakeri* (60). The functions of MC(s) during helminth infections have long been studied using MC(s)-deficient Kit^w^/Kit^W-v^ mice and employing MC(s) stabilizers (60). Using these approaches, it has been shown that MC(s)-deficient Kit^w^/Kit^W-v^ mice infected with *H. polygyrus* exhibited elevated intestinal worm burdens, reduced production of type 2 cytokines, as well as decreased serum levels of MC protease-1 (Mcpt1) (60). Further, treatment of mice with the MC(s) stabilizer, cromolyn sodium resulted in suppressed type 2 cytokine production and reduced Mcpt1 levels post-*H. polygyrus* infection (60). Furthermore, MC(s)-deficient Kit^w^/Kit^W-v^ mice also showed delayed expulsion of *Trichinella spiralis* (61) and studies have demonstrated that Mcpt1 and mast cell-derived IL-4 are required for optimal clearance of *T. spiralis* (52, 56, 62, 63). Collectively, these studies highlight an important role for MCs in promoting immunity to several helminth parasites (31, 62, 63). In contrast, by taking similar loss-of-function approaches, it has been shown that MC(s) do not appear to promote worm expulsion in the context of *Nippostrongylus brasiliensis* or *T. muris* infection, suggesting that the roles MC(s) play are parasite-specific (31, 60, 62, 64, 65).

## The contributions of hematopoietic stem/progenitors to host protection

It has traditionally been reported that the developmental pathways for red blood cells (RBCs) and MCs begin in the bone marrow with hematopoietic stem cells (HSCs), which can differentiate into a colony forming unit committed to the granulocyte, erythrocyte, monocyte, and megakaryocyte lineages (CFU-GEMM) (66) or follow the differentiation pathways of myeloid and lymphoid cells (39). Likely due to activation by circulating erythropoietin (EPO), CFU-GEMM can become burst-forming units-erythroid (BFU-E) (67) and thereby generate RBCs. In the case of MC development, instead of being influenced by EPO and entering erythropoiesis, the CFU-GEMM can be acted upon by stem-cell factor to develop down the granulocyte pathway (53). During this process, CFU-GEMMs increase their CD34 expression and become multipotent progenitors (MPP) (67). MPPs can then progress to become common myeloid progenitors (CMP) followed by granulocyte/monocyte progenitors (GMP) that can ultimately become a committed MC progenitor (MCp) (68–70). These MCps can then mature into MCs with heterogeneous properties depending on factors such as their tissue location (70). Additionally, emerging studies investigating MC and erythrocyte development now suggest that they may share more developmental similarities than previously appreciated.

Recent single cell-based work in humans and mice have determined that RBCs and MCs are directly linked and arise from a common progenitor cell (56, 57, 71, 72). Consistent with a link between these distinct lineages, a progenitor cell with dual MC and RBC potential was also identified in the context of a *T. spiralis* challenge (57). This erythrocyte/mast cell progenitor was defined by its expression of the metabolic enzyme carbonic anhydrase 1 (Car1) and was sufficient to promote type 2 cytokine responses and RBC development post-*T. spiralis* infection (56, 57). This work suggests that in addition to supporting protective immunity via promoting MC development, Car1-expressing progenitor cells may also combat helminth-induced wounding by supporting RBC development and thereby help to mitigate blood loss, a common feature of infection.

These studies complement earlier work that further suggest important roles for hematopoietic stem/progenitors (HSPCs) in promoting antihelminth immunity. The tissue-derived cytokines (IL25, IL33, and TSLP) can promote the populations expansion of other multipotent progenitor cells that have varied expression of CD34 and c-Kit. These specialized progenitors can develop into several innate immune (MCs, basophils, and macrophages) and thereby promote antihelminth immunity (31, 58, 73). After entering the periphery with an immature phenotype, helminth-mobilized HSPCs can undergo extramedullary hematopoiesis and support innate immune responses at the host parasite interface (31, 58, 73). Collectively, these studies demonstrate that the egress of HSPCs from the bone marrow is an important component of host protection (31, 56–58, 73).

## The contributions of basophils to antihelminth immunity

Although the contributions of basophils to antihelminth immunity and type 2 inflammation have long been studied, their diverse functions are still being elucidated. At baseline, basophils are extremely rare and represent the least prevalent granulocyte in the blood. However, peripheral basophilia is a hallmark of several helminth infections (74, 75). While basophil development has traditionally been reported to occur in the bone marrow, recent work also suggests that basophils can develop from mobilized progenitor cells that enter the periphery in the context of helminth-induced inflammation (75–77). Similar to MCs, basophils can produce robust amounts of effector molecules including type 2 cytokines (IL-4 and IL-13), histamines, platelet-activating factor, and lipid mediators (prostaglandins and leukotrienes) that allow them to promote worm clearance (75, 78). Additionally, their production of growth factors like amphiregulin and macrophage colony-stimulating factor are also thought to promote tissue reparative pathways (79, 80).

The most effective systems for studying basophil function have been genetic mouse models targeting the basophil-specific protease MCs protease 8 (Mcpt8) and basophil-specific IL-4 enhancer elements (75, 81). Using these systems, loss-of-function studies have indicated a non-redundant role for basophils in promoting worm expulsion following *T. spiralis*, *T. muris*, and *H. polygyrus* infections (82–84). However, basophil depletion had no effect on worm burdens following a primary infection with *N. brasiliensis* or *S. ratti* (85–87). Additionally, depleting basophils post-*Strongyloides venezuelensis* infection resulted in lower *S. venezuelensis* egg production, suggesting that basophils regulate parasite fitness (88). In summary, like many other innate immune cells, the functions of basophils appear to be highly parasite-specific.

As mentioned above, host protective responses to helminths involves both promoting worm expulsion and mitigating helminth-induced tissue damage. While loss-of-function studies targeting basophils revealed no effect in regulating *N. brasiliensis* worm burdens, additional work has now revealed that basophils depletion results in dysregulated lung inflammation. *N. brasiliensis*-induced ILC2 responses were found to be exaggerated in the absence of basophils, resulting in increased lung pathology and reduced pulmonary function (78). The inhibitory effect of basophils was mediated by neuro-immune interactions; the nature of these signals will be discussed in greater detail below. Conceptually, these studies suggest that basophils can also promote host protection by restricting helminth-induced inflammation and preventing excessive tissue damage.

## The contributions of eosinophils to antihelminth immunity

Similar to MCs and basophils, peripheral eosinophilia is a common feature of parasitic helminth infections (89). Eosinophils traffic to helminth-affected tissues where they are reported to contribute to worm killing and various aspects of tissue remodeling (90–93). Eosinophils produce numerous effector molecules including eosinophil-derived neurotoxin, major basic protein, and eosinophil peroxidase that can contribute to type 2 cytokine responses and simultaneously promote extracellular matrix deposition and wound healing (93, 94). Further, recent work also suggest that eosinophils can inhibit the mobility of parasitic larvae in an antibody-dependent manner (95).

While eosinophils can be recruited by both chemokines and cytokine alarmins (31, 96, 97), IL-5 produced by helminth-activated ILC2s and T_H_2 cells is a dominant regulator of infection-induced eosinophilia (98, 99). Consistent with these reports, mice lacking productive IL-5/IL-5R signaling are less able to mount an eosinophilic response and are less efficient at clearing *T. spiralis* (92, 98). Recent work has also showed that both mouse and human eosinophils can respond directly to parasite antigens isolated from *Strongyloides stercoralis* and *S. mansoni* respectively (100, 101). Although eosinophils produce numerous effector molecules, the mechanisms eosinophils employ to kill parasitic worms remain to be fully defined. It has been hypothesized that the release of chromatin and DNA extracellular traps may be one killing mechanism eosinophils use to combat large extracellular pathogens, but more work is required to support this hypothesis (102). Further, serum levels of eosinophil granular proteins are reported to be elevated in individuals infected with helminths such as *S. stercoralis*, indicating eosinophils may also activate and degranulate at distal sites (93). These data suggest that eosinophils may contribute to host protection beyond their roles in killing worms at the host parasite interface.

## Contributions of dendritic cells to antihelminth immunity

Dendritic cells (DCs), known for their professional antigen presenting cell (APC) capacities, are appreciated as important liaisons that bridge the gap between innate and adaptive immunity. As such, DCs are known for their pivotal roles in the recognition, capture, processing, and presentation of helminth-derived antigens to T cells (103–105). Many studies have reported that helminth ES products can activate DCs via toll-like receptor 2 (TLR2), TLR4, or C-type lectin receptors (103). Moreover, it has also been shown that helminth infections can promote non-classical DC maturation which is reported to dramatically influence T cell activation [reviewed in (103)]. For example, *T. spiralis* ES antigens and Glutathione-S-transferase can suppress DC maturation (106, 107) and *T. spiralis*-conditioned DCs can alleviate 2,4,6-trinitrobenzene sulfonic acid (TNBS)- induced colitis in mice (108). More recently, Ding et al. also reported that DCs stimulated by *T. spiralis* ES products were able to significantly inhibit tumor growth in H22 tumor-bearing mice (109). Interestingly, Connor and Webb et al. also found that DCs adopt a type 1 interferon (IFN-I) signature when stimulated with *S. mansoni* or *N. brasiliensis* antigens (110, 111). Of note, this IFN-I responsiveness was required for DCs to prime T_H_2 immune activation in these contexts (110, 111).

It is well established that conventional DCs (cDCs) can be subdivided into cDC1s and cDC2s that possess unique effector functions and abilities to polarize T cells (112). Specifically, cDC1s (CD8α^+^CD103^+^) are reported to specialize in antigen cross-presentation and promote T_H_1 cell development that supports immunity to intracellular pathogens. In contrast, cDC2s (CD4^+^CD11b^+^) that express interferon regulatory factor 4 (IRF4) specialize in presenting antigen to CD4^+^ T cells and possess a unique ability to promote T_H_2 or T_H_17 responses (112, 113). Given the plasticity of these DC subsets, it is perhaps not surprising that specialized DCs have also been reported to promote antihelminth responses. Cook et al., revealed that DC production of RELMα is required for optimal T_H_2 priming post-*S. mansoni* egg challenge (114). Moreover, T_H_2-inducing DCs expressing OX40 ligand, CD301b and programmed death ligand-2 (PDL2), are required for optimal T_H_2 cell development post-*N. brasiliensis* infection (115–117). More recently, Halim et al. showed that IRF4^+^CD11b^+^CD103^−^ DCs produce the T_H_2 cell-attracting chemokine CCL17 post-*N. brasiliensis* challenge (118). Studies by Mayer et al., also identified a role for IRF4-expressing DCs in priming T_H_2 cell responses following *S. mansoni* egg challenge (119).

Interestingly, CD11b^+^CD103^+^ DCs were shown to promote T_H_2 responses in the small intestine, while CD11b^+^CD103^-^ DCs appear to play similar roles in the colon, suggesting functional-specificity for DCs in different anatomical compartments (119). Collectively, these studies demonstrate that highly specialized DC subsets play important roles in promoting antihelminth immunity and suggest that CD103 expression may dictate the tissue specificity of these APCs.

Unlike conventional DCs, the roles of plasmacytoid DCs (pDCs) in antihelminth immunity remain less defined. While studies suggest that pDCs are dispensable for hepatic T_H_2 responses during acute *S. mansoni* infection (23, 120), other reports suggest that pDCs are required for optimal T_H_2 cytokine production in response to *S. mansoni* eggs in the intestinal-draining mesenteric lymph nodes (120). Furthermore, pDC depletion at chronic stages of infection resulted in increased hepatic and splenic pathology as well as suboptimal T_H_2 cytokine production in the liver. However, further studies are needed to better define the role pDCs play in promoting antihelminth immunity and regulating tissue pathology.

## The contributions of neutrophils to antihelminth immunity

Although neutrophils are best known for their roles in antiviral and antibacterial immunity, recent studies have begun to define a role for these dynamic cells in the context of type 2 inflammation (121–123). For instance, recent reports have demonstrated that neutrophils can inhibit the mobility of *S. ratti* larvae via their release of myeloperoxidase and matrix metalloproteinase-9. Additionally, studies have also highlighted that neutrophils are recruited to the lung post-*N. brasiliensis* infection where they contribute to tissue damage and hemorrhaging (124). It is reported that *N. brasiliensis*-induced neutrophils are recruited by local production of IL-17A from activated γδT cells in response to chitinase-like proteins (CLPs), such as Ym1 (121, 122). Additionally, it has been shown that soluble extracts from *S. stercoralis* promote neutrophil recruitment through CXCR2, rather than IL-17, suggesting helminth-induced neutrophils may be regulated by distinct signals (125). Even though γδ^+^ intraepithelial lymphocyte populations are found to be expanded post-*T. muris* and -*T. spiralis* infection (126, 127), whether neutrophil recruitment is initiated in the context of these infections remains unknown.

Importantly, work by Chen et al. showed that neutrophils sort-purified from the lungs of *N. brasiliensis*-infected mice had a distinct transcriptional signature compared to lipopolysaccharides-activated neutrophils (121). One dominant feature of *N. brasiliensis* -induced neutrophils was their increased expression of the type 2 cytokine IL-13, prompting them to be named N2 neutrophils. Moreover, it was shown that neutrophil-derived IL-13 promoted M2 macrophage polarization (121). In addition to IL-13, neutrophils have also been shown to release neutrophil extracellular DNA traps (NETs) to promote antihelminth immunity. Specifically, NETs were found to be released upon contact with *S. stercoralis* larvae (128). Although the NETs failed to kill the larvae, they helped immobilize parasites (129) and may assist with “starving” the worms by trapping them in a nutrient-deficient microenvironment similar to what has recently been shown by macrophages (130). Further, Bouchery et al., showed that NETs released following an *N. brasiliensis* challenge can directly impair larval viability and the parasites combat this response by secreting DNAse II (123). The ability of helminth-derived products to inhibit NET formation was also shown by Chauhan et al. that demonstrated that *Mesocestoides corti* ES products were sufficient to inhibit NET formation in the context of bacterial peritonitis (131). Moreover, it was reported that neutrophils and eosinophils require myeloperoxidase and major basic protein to kill *S. stercoralis* larvae *in vitro* (93). Therefore, it is possible that the NETs immobilize the parasites and thereby maximize the parasitic exposure to antihelminth products secreted by activated granulocytes and macrophages. Taken together, these studies suggest that neutrophils promote host protective responses to helminths via a variety of effector functions, however, further work is required to better understand how parasite-specific these responses are, and the full range of effector functions neutrophils employ in these contexts.

## The contributions of monocytes and macrophages to antihelminth immunity

It is well established that the IL-4 and/or IL-13-mediated activation of macrophages results in their polarization to what has traditionally been termed as an alternatively activated or M2 phenotype. The importance of M2s and their roles in promoting host protection to helminths is well established and has been extensively reviewed elsewhere (132–135). Therefore, we will briefly highlight this impressive body of literature and largely focus our discussion on recent studies describing heterogeneity within tissue-specific macrophage responses.

The induction of M2s has been demonstrated in the context of numerous helminth infections including *N. brasiliensis*, *S. mansoni*, *H. polygyrus*, *Taenia crassiceps*, *T. spiralis*, *Fasciola hepatica*, *Ascaris suum*, *and filarial* parasites (136–143). While IL-4 and/or IL-13 produced by various myeloid and lymphoid cells are known to promote M2 responses (124, 132, 137, 144–146), additional factors can also facilitate M2 activation including antibodies (IgG), collectins (surfactant protein A & D), complement components, helminth ES products (34, 132), TLR and CLR ligands, macrophage migration inhibitory factor (MIF), macrophage-derived protease inhibitor (serpinB2), cytokine alarmins, and metabolic cues (vitamin A) (58, 147–150). M2s are known to promote host protection via several mechanisms including the release of effector molecules and chemokines to promote type 2 responses, directly or indirectly killing parasitic larvae, promoting wound healing by stimulating collagen deposition, and angiogenesis (114, 132, 133, 135, 150, 151).

Macrophages reside in every organ and mucosal surface and exhibit distinct phenotypes and effector functions depending on their tissue-specific niche (132). Importantly, recent studies have also revealed that macrophages are specially programmed to operate in an organ-specific manner (152–157). Tissue-resident macrophages (TRMs) that are derived from embryonic precursors, seed the tissues during early stages of development and are tailored to perform tissue-specific tasks. While TRMs have been shown to proliferate in the context of inflammation (158), additional monocyte-derived macrophages can enter these niches to supplement TRM responses. Upon entering the tissue, monocyte-derived macrophages receive tissue-specific signals and begin to acquire a TRM-like phenotype (115, 159, 160). These studies strongly suggest that M2 responses occurring post-helminth infection are comprised of a heterogenous group of cells. Additionally, recent work has shown that TRMs and monocyte-derived macrophages can perform unique antihelminth functions. For instance, monocyte-derived alveolar macrophages induced post-*N. brasiliensis* infection are more effective at killing parasites than TRMs. The heightened ability of monocyte-derived alveolar macrophages to kill parasitic larvae was mediated by their enhanced expression of arginase 1 which allowed them to deplete local arginine (130). Further, by comparing macrophages from *S. mansoni* or *Litomosoides sigmodontis*-infected mice, along with IL-4 and anti-IL-4 antibody complexes (IL-4c) and thioglycolate-treated mice, Gundra et al. reported that monocyte-derived macrophages are more immunoregulatory than TRMs (161). Moreover, additional work identified that vitamin A was essential to instruct the tissue programming of macrophages from a monocyte-derived phenotype to a more TRM-like phenotype (149). Collectively, these emerging studies suggest that antihelminth macrophage responses are more heterogeneous than previously appreciated. Further, this important work suggests that several factors dictate how M2 macrophages are regulated, including the nature of the parasite, the origin of the cells (monocyte-derived versus tissue-resident) and the signals they receive from the tissue microenvironment.

## Contributions of myeloid-derived suppressor cells to antihelminth immunity

Myeloid-derived suppressor cells (MDSCs) were initially described in cancer for their ability to inhibit anti-tumor T cells but have subsequently been appreciated for their immunosuppressive roles in response to pathogens including helminth (162, 163). MDSCs are a heterogenous group of cells that can be divided into two major groups, granulocytic/polymorphornuclear MDSCs (PMN-MDSCs, Gr1^+^CD11b^+^Ly6G^+^Ly6C^lo^) and monocytic MDSCs (M-MDSCs, Gr1^+^CD11b^+^Ly6G^-^Ly6C^hi^). Infections with *S. mansoni, S. japonicum, T. crassicepts*, *Brugia malayi*, *N. brasiliensis*, and *H. polygyrus* have all been shown to induce MDSCs that are thought to play important immunoregulatory roles (162). The diverse roles MDSCs play in regulating helminth-induced inflammation is discussed in great depth by Stevenson et al. in a recent review article and therefore will not be discussed in depth here (162).

## The contributions of innate lymphoid cells to antihelminth immunity

Innate lymphoid cells (ILCs) are tissue-resident cells that lack adaptive antigen receptors and are considered the non-specific counterparts of T lymphocytes. They reside in various tissues including the lung, intestine, mesenteric fat associated lymphoid cluster, liver, skin, and kidney, and are appreciated for their pivotal roles in promoting immunity to bacteria, viruses, and parasitic infections. ILCs are classified into 5 distinct subsets – nature killer (NK) cells, ILC1s, ILC2s, ILC3s, and lymphoid tissue inducer cells based on their developmental origins, transcriptional and surface marker phenotypes, as well as functional differences (164). Importantly, various subsets of ILCs have been shown to play diverse roles in regulating antihelminth immunity which will be highlighted in the below sections.

NK cells have been shown to accumulate during the early phases of *H. polygyrus* infection in an IFNγ receptor-dependent manner where they are thought to promote tissue protection (165). NK cells have also been shown to be activated following *S. japonicum* infection*, S. mansoni* infection, and *S. mansoni* egg challenge (166, 167). Consistent with animal models, human studies have also indicated that NK cells appear to respond to helminths (168, 169). However, future studies are needed to better elucidate the functions of NK cells in these contexts.

ILC2s are well described for their ability to respond to cytokine alarmins (IL-25, TSLP, and IL-33) and as such become rapidly activated in the context of helminth infections (34, 170, 171). In addition to alarmins, ILC2s are also regulated by type 2 cytokines (IL-4 and IL-9) and inflammatory lipid mediators that are hallmarks of type 2 inflammation (172, 173). Once activated, ILC2s are reported to produce robust levels of IL-5 and IL-13 and thereby support the population expansion and recruitment of eosinophils, the M2 polarization of macrophages, and mucus production by goblet cells (28, 32, 33, 82, 121, 124, 132, 170, 171, 174, 175). Helminth activated ILC2s have also been shown to produce IL-4 and IL-9, although these cytokines appear to be less prominent (171, 176). These studies are excessively discussed in review articles by Herbert et al., Bouchery et al., and Miller et al. (177–179). Additionally, growing evidence suggests that the antihelminth functions of ILC2s are regulated, in part, by neuron-derived signals. The importance of these pathways will be discussed in greater depth below.

## Neuro-immune communication during helminth infections

The central nervous system is responsible for maintaining homeostasis during steady state conditions and in the context of infection and inflammation (180, 181). To accomplish this, complicated cellular and molecular networks have been established to allow highly coordinated communication between the nervous and immune system to occur (75, 78, 182–186). Given the intricate relationship helminths have developed with their mammalian hosts, it is not surprising that many of these pathways play critical roles in promoting host protection and regulating antihelminth immunity. Emerging studies have significantly advanced our understanding of these intricate networks and have encouraged more interdisciplinary collaborations to better understand neuro-immune interactions in the context of helminth-induced inflammation. The following section will highlight pathways that are known, or likely, to regulate neuro-immune communication during helminth infections. Additionally, we will comment on the need for future studies to further determine how these pathways operate in response to this diverse class of pathogens.

**Figure 2:**
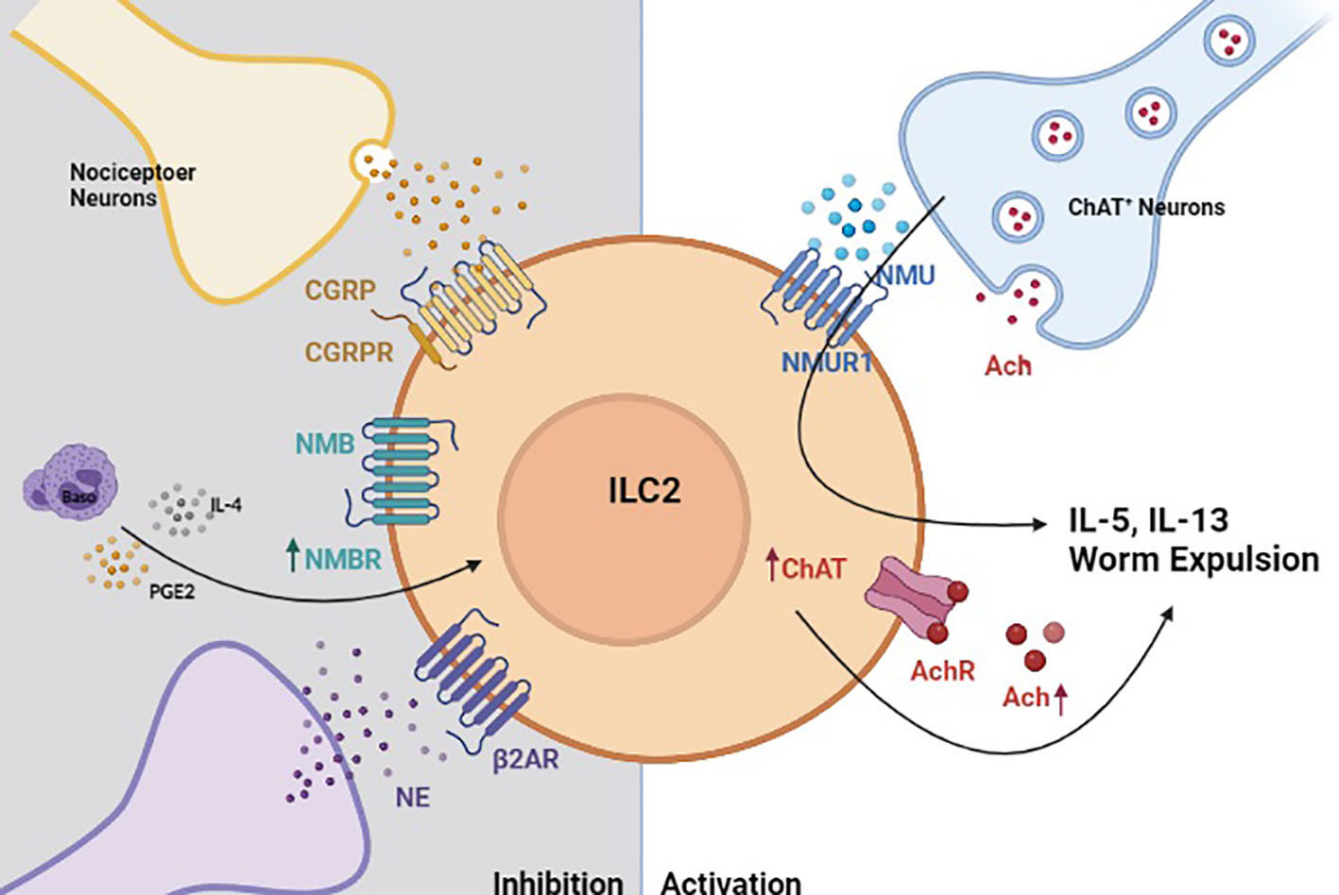
Neuro-Immune crosstalk regulates helminth-induced inflammation. In the context of helminth-induced inflammation, group 2 innate lymphoid cells (ILC2s) are activated by neuromedin U (NMU) that is released by choline acetyltransferase positive (ChAT^+^) neurons (185). Activated ILC2s also upregulate ChAT to promote acetylcholine biosynthesis, which serves to further amplify their production of type 2 cytokines (184). Helminth-activated ILC2s are also restricted by neuron-derived signals. Calcitonin gene-related peptide (CGRP), neuromedin B (NMB) and sympathetic neuron-derived norepinephrine (NE) inhibit ILC2 response in a manner that properly regulated helminth-induced inflammation (78, 182, 183).

Novel transgenic animal models, precise activation techniques (chemogenetics and optogenetics), and other emerging technologies have greatly facilitated our ability to interrogate the pathways that regulate rare immune cells located within helminth-affected tissues (23, 75, 186). These intricate studies have recently revealed several neuron-derived signals that regulate ILC2 responses post-*N. brasiliensis* infection (Summarized in **Figure 2**). For instance, the neuropeptide neuromedin U (NMU) was recently shown to directly activate ILC2s through its receptor NMUR1 to drive antiparasitic immunity post-*N. brasiliensis* infection. In the intestine, a subset of enteric neurons express NMU (185, 187) and colocalize with ILC2s. NMU induces ILC2 proliferation and production of type 2 cytokines, such as IL-5, IL-9, and IL-13. Additionally, Chu et al. found that activated ILC2s upregulate choline acetyltransferase to generate more acetylcholine (Ach) following *N. brasiliensis* infection or treatment with cytokine alarmins (184). Importantly, Ach was sufficient to promote ILC2 cytokine production and their expulsion of *N. brasiliensis* (184). Another neuropeptide calcitonin gene-related peptide (CGRP), expressed by nociceptor neurons was shown to inhibit ILC2 activation and thereby limit antihelminth responses (183). Similarly, neuromedin B (NMB) was also shown to restrict ILC2 activation in the lungs as part of a basophils-dependent feedback loop following *N. brasiliensis* infection (78). Interrupting NMB-NMBR interactions was also shown to result in substantially increased lung pathology and reduced lung function post-infection, suggesting that its inhibitory effects are required to maintain tissue integrity. This work also showed that prostaglandin E (PGE), one of several basophil-derived lipid mediators, can stimulate NMBR expression on ILC2s and thereby prime them for NMB-mediated inhibition (**Figure 1**). Finally, sympathetic neurons can also inhibit ILC2 responses and helminth clearance by activating beta-2 adrenergic receptor, which are expressed by ILC2s (182).

In addition to ILC2s, MCs have long been observed to be anatomically and functionally associated with neurons and neuronal processes throughout the body. Additionally, neural regulation of MCs has been described in disease models of atopic dermatitis, allergic asthma, and chronic obstructive pulmonary disease (160, 188). MCs express a variety of neuron-related receptors, transmitters and peptides, such as Ach, substance P, and CGRP among others (189). Recent studies have also highlighted a pivotal role of Mas-related G-protein-coupled receptors (Mrgprs) in neuro-immune crosstalk (188, 190). Mrgprs are highly expressed in MCs and are reported to mediate MC degranulation in an IgE-independent manner (191, 192). More recently, Arifuzzaman et al., found that cutaneous bacterial infection can activate MC activation through an MrgprB2/MRGPRX2-mediated pathway, which leads to enhanced recruitment of neutrophils and wound-healing CD301b^+^ DCs (193), both cell types that are known to promote antihelminth immunity. Zhang et al. also reported that MrgprD-expressing neurons can suppress MC hyperresponsiveness and skin inflammation by releasing glutamate (194). Interestingly, one recent study showed that tick peptides can cause histamine-independent itch, by directly activating MrgprC11/MRGPRX1 on the dorsal root ganglion and MrgprB2/MRGPRX2 on MCs (195). Furthermore, other non-helminth models have revealed that MCs can stimulate itch sensory and nociceptor neurons to promote itch sensation and type 2 inflammation in the skin (168, 196, 197). Collectively, these studies highlight important roles for MCs in mediating neuro-immune communication at barrier surface. The ability of MCs and other tissue-resident cell types such as macrophages to participate in neuro-immune mechanisms that regulate antihelminth responses is an active and exciting area of study that will further inform our understanding of how inflammation is regulated in the tissue microenvironment. Further, whether neuro-immune communication is bidirectional and also involves helminth-activated immune cells regulating the functions of the central nervous system is also an area of great interest.

## Summary

A robust body of literature has highlighted the important functions of various innate immune cells in regulating host protective responses to helminths. Further, it is now appreciated that many of these responses are regulated in both parasite- and tissue-specific manners. Given the ancient and complex relationship between helminth and their mammalian hosts, studying these infections provides a unique lens into the factors that regulate tissue-specific immunity. This work has begun to highlight the importance of the peripheral nervous system in positively or negatively regulating helminth-induced inflammation. The ability of neuron-derived signals to amplify or restrict antihelminth responses may allow them to tailor the inflammation to promote optimal outcomes (inflammation strong enough to promote worm expulsion, but tightly regulate to prevent excessive tissue damage). However, future studies are required to better understand how these seemingly opposing signals operate post-infection and to determine whether other tissue-resident cell types such MCs, TRM, and monocyte-derived macrophages are similarly involved in these processes. Gaining a better understanding of these pathways may inform therapeutic strategies to treat a myriad of inflammatory conditions and reveal more efficient ways to treat tissue-specific pathology.

## Author contributions

JP and MCS contributed to the generation of the manuscript, in terms of material development, content creation, and proofreading. HGF and CMH contributed to the generation of certain paragraphs, related literature search, and proof reading of the manuscript. All authors contributed to the article and approved the submitted version.

## Conflict of interest

MCS is the founder and president of NemaGen Discoveries.

The remaining authors declare that the research was conducted in the absence of any commercial or financial relationships that could be construed as a potential conflict of interest.

## Publisher’s note

All claims expressed in this article are solely those of the authors and do not necessarily represent those of their affiliated organizations, or those of the publisher, the editors and the reviewers. Any product that may be evaluated in this article, or claim that may be made by its manufacturer, is not guaranteed or endorsed by the publisher.
